# Fear of Reprisal and Change Agency in the Public Health and Social Service System: Protocol for a Sequential Mixed Methods Study

**DOI:** 10.2196/48400

**Published:** 2023-09-21

**Authors:** Annie Carrier, François Bolduc, Nathalie Delli-Colli, Finn Makela, Anne Hudon, Marie-Eve Caty, Arnaud Duhoux, Michaël Beaudoin

**Affiliations:** 1 School of Rehabilitation Faculty of Medicine and Health Sciences Université de Sherbrooke Sherbrooke, QC Canada; 2 Research Center on Aging University Integrated Health and Social Services Centre of the Eastern Townships – Sherbrooke University Hospital Sherbrooke, QC Canada; 3 Department of Industrial Relations Faculty of Social Sciences Université Laval Quebec, QC Canada; 4 Interuniversity Research Centre on Globalization and Work Université de Montréal Montreal, QC Canada; 5 School of Social Work Faculty of Arts and Humanities Université de Sherbrooke Sherbrooke, QC Canada; 6 Faculty of Law Université de Sherbrooke Sherbrooke, QC Canada; 7 School of Rehabilitation Faculty of Medicine Université de Montréal Montreal, QC Canada; 8 Centre for Interdisciplinary Research in Rehabilitation of Greater Montreal University Integrated Health and Social Services of the South-Center-of-Montreal-Island Montreal, QC Canada; 9 Research Center for Ethics Université de Montréal Montreal, QC Canada; 10 Department of Speech-Language Pathology Université du Québec à Trois-Rivières Trois-Rivières, QC Canada; 11 Faculty of Nursing Université de Montréal Montreal, QC Canada; 12 Research Centre of the Integrated Health and Social Services Centre of Laval Laval, QC Canada

**Keywords:** change agency, fear, professional practice, retaliation, social justice

## Abstract

**Background:**

Since they are key witnesses to the systemic difficulties and social inequities experienced by vulnerable patients, health and social service (HSS) professionals and clinical managers must act as change agents. Using their expertise to achieve greater social justice, change agents employ a wide range of actions that span a continuum from the clinical (microsystem) to the societal (macrosystem) sphere and involve actors inside and outside the HSS system. Typically, however, clinical professionals and managers act in a circumscribed manner, that is, within the clinical sphere and with patients and colleagues. Among the hypotheses explaining this reduced scope of action is the fear of reprisal. Little is known about the prevalence of this fear and its complex dynamics.

**Objective:**

The overall aim is to gain a better understanding of the complex dynamic process leading to clinical professionals’ and managers’ fear of reprisal in their change agent actions and senior administrators’ and managers’ determination of wrongdoing. The objectives are (1) to estimate the prevalence of fear of reprisal among clinical professionals and managers; (2) to identify the factors involved in (a) the emergence of this fear among clinical professionals and managers, and (b) the determination of wrongdoing by senior administrators and managers; (3) to describe the process of emergence of (a) the fear of reprisal among clinical professionals and managers, and (b) the determination of wrongdoing by senior administrators and managers; and (4) to document the legal and ethical issues associated with the factors identified (objective 2) and the processes described (objective 3).

**Methods:**

Based on the Exit, Voice, Loyalty, Neglect model, a 3-part sequential mixed methods design will include (1) a web-based survey (objective 1), (2) a qualitative grounded theory design (objectives 2 and 3), and (3) legal and ethical analysis (objective 4). Survey: 77,794 clinical professionals or clinical managers working in the Québec public HSS system will be contacted via email. Data will be analyzed using descriptive statistics. Grounded theory design: for each of the 3 types of participants (clinical professionals, clinical managers, and senior administrators and managers), a theoretical sample of 15 to 30 people will be selected via various strategies. Data will be independently analyzed using constant comparison process. Legal and ethical analysis: situations described by participants will be analyzed using, respectively, applicable legislation and jurisprudence and 2 ethical models.

**Results:**

This ongoing study began in June 2022 and is scheduled for completion by March 2027.

**Conclusions:**

Instead of acting, fear of reprisal could induce clinical professionals to tolerate situations that run counter to their social justice values. To ensure they use their capacities for serving a population that is or could become vulnerable, it is important to know the prevalence of the fear of reprisal and gain a better understanding of its complex dynamics.

**International Registered Report Identifier (IRRID):**

PRR1-10.2196/48400

## Introduction

As they are in regular contact with patients who are vulnerable or at risk of becoming vulnerable, health and social services (HSS) professionals are often the first to see the systemic difficulties and social inequalities these patients encounter. These professionals are then called upon to act to defend the rights and promote the social inclusion of these patients, in other words, to assume the role of change agent [1-6]. From both a professional [1-6] and an ethical [7] perspective, this role is not optional, it is an obligation. Clinical managers (eg, department heads and coordinators), who are usually HSS professionals [8], also take on this role. In times of uncertainty (pandemic and disaster), it is all the more critical to act as a change agent because of increasing inequities, including in HSS [9,10]. The COVID-19 pandemic revealed or exacerbated existing social and health inequities in populations at risk of increased vulnerability, including seniors and people with disabilities, chronic illnesses, or mental health issues [9,10]. Using their expertise and influence to achieve greater social justice [7], change agents employ a wide range of actions that span a continuum from the clinical (microsystem) to the societal (macrosystem) sphere and involve actors both within and outside the HSS system [11]. To increase social inclusion and justice for people with heightened vulnerability, however, the clinical actions typically mobilized (such as advocating for a patient’s choices in a team meeting) are not enough [12]. In light of the systemic issues that persist in HSS, including ableism, ageism, and racism [13], social actions such as advocating to policy-making bodies, serving in an official capacity within an advocacy organization, or speaking out in public [11] are also necessary to stimulate positive systemic change.

Typically, HSS professionals and clinical managers act as change agents in a circumscribed manner, at the clinical level and with patients and colleagues. For example, in a quantitative cross-sectional study [14], the change agency practice of occupational therapist practitioners and clinical managers included little social action (such as public speaking) and rarely targeted higher level actors in their institution (eg, senior administrators or senior managers) or outside it (eg, senior government officials and elected officials). One of the hypotheses suggested to explain the lack of action outside the clinical sphere is the fear of reprisal. This fear emanates from a risk of retaliation, whether this risk is real or not [15]. Reprisals are “action[s] taken by an individual or group to inflict physical, economic, or other harm in response to an action taken by another” (free translation [16]). They include but are not limited to formal sanctions. Instead of taking action, fear of reprisal may lead clinical professionals and managers to tolerate situations that run counter to their social justice values [12]. In an interpretive qualitative study with change agents recognized as such by their peers, participants reported that having a system that “supports” the agency of professionals is one of the essential elements for carrying out change actions [17]. Thus, to ensure they are using their capacities as agents to serve a population that is or could become vulnerable, HSS professionals and clinical managers should not fear reprisals for their actions to improve social inclusion and justice for their patients; rather, they should feel supported in their change efforts.

Some authors [14,18] suggest that a fear of negative repercussions may be rooted in part in how professionals and their managers interpret the duty of loyalty to their employer [19]. Other potential causes associated with the duty of loyalty include the confidentiality agreement that new employees must sign in some HSS settings, disciplinary proceedings for professional misconduct (eg, Carolyn Strom in Saskatchewan [20]), and disciplinary action taken by the employer (eg, nurses suspended without pay [21]). To our knowledge, no empirical study has specifically examined the fear of reprisal associated with change agents’ actions, or sought to understand when and under what conditions this fear arises. Some studies that focused on the change actions of professional practitioners and managers did, however, document part of this reality. In particular, an exploratory qualitative study [22] examined the actions of 39 nurses who were politically engaged in their hospital practice setting. Fear of retaliation by colleagues and managers was reported as a barrier to change actions in the clinical setting. Among the types of retaliation used, participants described “various forms of harassment, violence (often public), confrontation, intimidation or threats, loss of privilege, and abandonment by colleagues...increased monitoring...sudden deterioration of relationships with their superiors, ongoing tensions, and more frequent sanctions such as ‘going to [the superior’s] office’... [use of] disciplinary notes, various forms of coercion, such as suspensions and dismissal” [22]. Participants spoke of fear-based management to explain their reluctance to voice their opinions within the HSS system. By analyzing 597 anonymous whistleblower reports from outside the HSS system at the beginning of the COVID-19 pandemic, Perron et al [18] were also able to identify retaliation against nurses, including intimidation, threats, and sanctions. These 2 studies shed light on nurses’ perceptions of potential or actual retaliation for acting as change agents. However, it is not clear if this view is shared by all HSS professionals and clinical managers. Moreover, since they are also concerned about a fear of reprisal, the perspective of senior administrators and senior managers regarding what constitutes an improper change agent action must be considered. To date, nothing is known about this perspective.

In “professionalizing” HSS university programs, professional competencies, including those related to the change agent role, are being developed and fostered by a longitudinal, gradual, and explicit deployment of learning activities [23]. This deployment takes place in authentic professional situations [24] involving high ecological validity and increasing complexity [23], drawing on transformational learning [25] and critical consciousness [26], and combined with formative (eg, providing feedback) and summative assessments of competencies through programmatic or longitudinal approaches [27]. Both ecological validity and increasing complexity optimize the preparation of future professionals for their actual practice conditions. This preparation is associated with an increased sense of competence in practice [28] and consequently a greater motivation to act [29]. Further, by increasing their critical awareness [30], authentic educational experiences open up learners to professional transformation [31,32] and allow for reflexivity [33], which may assist in developing skills leading to patient advocacy and participation in social justice initiatives in partnership with community stakeholders. To ensure the ecological validity and increasing complexity of authentic educational activities related to the change agent role in HSS university programs, it is important to gain a better understanding of when and under what conditions fear of reprisal and determination of wrongdoing occur, respectively, in professional practitioners and clinical managers and in senior administrators and senior managers. Knowledge of these processes is crucial to better prepare future HSS professionals to incorporate the change agent role in their practices, and hence support high quality, compassionate, and equitable health and social care. Eventually, professionals trained in this way will help to increase social inclusion and justice for people with heightened vulnerability.

## Methods

### Objectives and Design

The aim of this study is to gain a better understanding of the complex dynamic process leading to clinical professionals’ and clinical managers’ fear of reprisal in their change agent actions and senior administrators’ and senior managers’ determination of wrongdoing. More specifically, the objectives are (1) to estimate the prevalence of fear of reprisal among clinical professionals and managers, overall and by profession and practice setting; (2) to identify the factors involved in (a) the emergence of this fear among clinical professionals and managers, and (b) the determination of wrongdoing by senior administrators and managers; (3) to describe the process of emergence of (a) the fear of reprisal among clinical professionals and managers, and (b) the determination of wrongdoing by senior administrators and managers; and (4) to document the legal and ethical issues associated with the factors identified (objective 2) and the processes described (objective 3).

We will use a 3-part sequential mixed methods design [34]. In the first component, a survey, the prevalence of fear of retaliation in the population under study, that is, clinical professionals and managers in HSS in Québec (Canada), will be estimated (objective 1). This type of design has already been used successfully to estimate the prevalence of this fear among Canadian workers according to different types of occupational health and safety advocacy actions [35]. The aim of the second component, a qualitative grounded theory design, will be to identify the factors involved in the emergence of this fear among clinical professionals and managers and in the determination of a wrongful action by senior administrators and managers in HSS in Québec (objective 2) as well as to describe the process of emergence of this fear among clinical professionals and managers and of this determination by senior administrators and managers (objective 3). Finally, in the third component, a legal and ethical analysis, the legal and ethical issues associated with the factors identified and the processes described will be documented (objective 4). Thus for components 2 and 3, there will be 2 study populations in HSS in Québec: clinical professionals and clinical managers, on the one hand, and senior administrators and senior managers, on the other.

### Theoretical Background

This study is based on the *Exit, Voice, Loyalty, Neglect* (EVLN) model [36], which presents 4 possible employee responses to unsatisfactory working conditions. This model is relevant to this study because professionals’ ability to act to improve social inclusion and justice for their patients is a function of their practice and thus of their working conditions. In the EVLN model, exit induces the professional to leave his or her job while voice leads him or her to speak up to improve the situation. Loyalty reflects his or her decision to wait. Finally, in neglect, the professional gives up trying to improve the situation and disengages or obstructs. The concept of interest here, namely fear, can be met with silence (nonaction; [37]). Depending on the reason for the professional’s “silence” [38], the silence will fall under loyalty (expectation) or negligence (disengagement). For example, as a strategy or due to resignation or fear, the professional may choose not to act (remain silent). The nature of silence can thus vary according to the characteristics of the situation and the moment [39]. Examples of characteristics and moments are the intensity and immediacy of the fear and the person with whom the professional is dealing (eg, senior manager). This finding is independent of the status of the professional (could be a department head); relatively high status workers are affected [40]. Even among workers hierarchically equivalent to clinical managers, actions may vary depending on the severity of retaliation, the coping strategy chosen, the relative organizational statuses of those involved, and the person’s gender. All of these contextual elements will be considered in the development of data collection tools for components 1 and 2. The EVLN model will also enrich the interpretation of the results of the 3 components.

### Study Components, Data Collection, and Analysis

#### Component 1: Survey

##### Overview

Given the common challenges they face, such as labor shortages [41], waiting lists, and wait times [42-44], as well as the at-risk vulnerable populations they serve, nurses, occupational therapists, physical therapists, social workers, and speech therapists working in the public HSS system will be the target population. In fact, these challenges and patient populations may increase the likelihood of acting or intending to act as change agents. To estimate the prevalence of fear of reprisal (objective 1), we will survey this entire population using a web-based self-administered questionnaire that we will develop.

##### Development, Validation, and Pretesting of the Questionnaire

To develop the questionnaire, we will use 3 alternative sources of information combined with the EVLN model. This will give us recent examples of situations involving fear of reprisal that are highly relevant to our target population. *Source 1:* we will conduct a review of papers citing fear of reprisal by HSS professionals in the Québec media from January 2015 (last HSS reform) to May 2022 using 2 databases: Eureka and the Canadian Business & Current Affairs Database. The former contains papers from Canadian news media, including periodicals, newspapers, and television and radio transcripts; the latter contains news stories, and radio and television transcripts. *Source 2:* We will collate case law from employment and professional tribunals concerning formal sanctions against HSS professionals and clinical managers from January 2015 to May 2022 from the 2 leading legal databases in Québec, CanLII, and Soquij. This source will provide examples of situations that may cause concern. *Source 3:* We will conduct 4 open-ended exploratory interviews, 2 with 2 union representatives and 2 with 2 association representatives, about their experiences with the relevant situations. For all 3 data sources, we will conduct a summative content analysis [45]. This type of analysis leads to an interpretation of the contextual meaning of specific terms or types of content. Supported by the EVLN model, we will then triangulate the distinctive elements of the reported situations, including change agency actions and manifestations of fear of reprisal. Based on these elements, we will develop survey questions focusing on change agent actions that were considered or carried out and the occurrence of fear of reprisal for these actions. The questionnaire will consist mainly of Likert scale questions (4 levels; from 0: not at all, to 3: completely) and closed multiple choice questions. For example, for actions, 1 question could be: “I have thought about making a claim or I made a claim to a manager regarding a situation of concern for my patients.” For the associated fear, 2 questions could be “When I thought about advocating or when I advocated to a manager about a concern for my patient, I feared retaliation” and “If you checked 1 to 3, choose which of the following retaliations you feared.*”* The Delphi method will be used to ensure content and face validity of the questionnaire [46,47]. Further, 10 content experts from various fields (law, management, industrial relations, and HSS) will be consulted during (content validity) and after (face validity) construction of the questionnaire. This systematic method of consultation is particularly useful when empirical knowledge is poor [48]. The consultation will be conducted by email and in successive rounds until a consensus of 80% (n=8) of experts for each question is reached. To characterize the profile of participants, a sociodemographic section (eg, occupation, background, and years of experience) will be added to the validated questionnaire as well as an invitation to participate in component 2 of this study. The questionnaire will then be pretested with 5 graduate students in health or management who have experience as a health professional or clinical manager, respectively.

##### Data Collection

As recommended by Dillman et al [48], the entire target population will be contacted. Our connections and credibility in the professional community (1 researcher from each profession) will facilitate recruitment. To be recruited, individuals will need to hold a professional or clinical management position, be a member of 1 of the 5 relevant professions, understand French, and have at least 6 months’ experience in the public HSS system. From their membership lists, 2 unions (one representing nurses and the other representing occupational therapists, physical therapists, social workers, and speech therapists) will send an email invitation to participate in this study and a link to the survey hosted on LimeSurvey (LimeSurvey GmbH). This strategy will also reach clinical managers. A total of 77,794 clinical professionals or clinical managers working in the public HSS system will be reached: 4125 occupational therapists, 2142 speech-language pathologists, 8895 social workers, 2489 physiotherapists (O Dallaire-Turmel, personal communication, August 13, 2021), and 60,143 nurses [8]. Considering the participation rate for recent surveys of the target population (eg, references [49,50]), we estimate that we will obtain a minimum participation rate between 30% (n=23,338)and 45% (n=35,007).

##### Analyses

To estimate the prevalence of fear (objective 1) and describe the participants, descriptive statistics (means, SD, frequencies, and percentages) will be used, overall by type of participant, and then by setting, profession, and position (professional or managerial). The data will be analyzed in Excel (Microsoft Corp) and SPSS (IBM Corp) and the analyses will be validated by all team members. The results will shed light on the elements to be studied in greater depth in component 2.

#### Component 2: Qualitative Grounded Theory

##### Overview

We will use a qualitative grounded theory design based on a constructivist perspective [51] with 3 types of participants: clinical professionals, clinical managers (department heads, coordinators), and senior administrators and managers. This type of design enables the exploration and definition of a little-known process (fear of reprisal and determination of reprehensible change agent actions) by taking into account all of the factors involved.

##### Recruitment and Sampling

To recruit our 3 types of participants, we will use three main recruitment strategies: (1) contact with component 1 participants who have indicated an interest in participating in component 2 (clinical professionals and clinical managers); (2) an email invitation from the *Association des gestionnaires des établissements de santé et de services sociaux* (managers) and the *Association des cadres supérieurs de la santé et des services sociaux* (senior managers); and (3) word of mouth through participants already recruited (clinical professionals, clinical managers, and senior administrators). *Association des gestionnaires des établissements de santé et de services sociaux* represents almost three-quarters of all managers in the HSS system [52] and all senior administrators and senior managers automatically become (unless voided) members of *Association des cadres supérieurs de la santé et des services sociaux* [53]. For clinical professionals and managers to participate in this study, the same inclusion criteria will apply as in component 1. In addition, they must have experienced a situation in which their change agency’s actions, either planned or carried out, led to a fear of reprisal. For managers to be included, they will have to hold a management position, understand and speak French, and have at least 6 months’ managerial experience in the public HSS system. For individuals recruited by convenience sampling and for each type of participant, we will then use a theoretical sampling technique to vary the heterogeneity of the personal characteristics and situations experienced by the participants. This will be done in successive phases with 2 or 3 participants, according to criteria guided by data analysis needs [54]. We estimate that a theoretical sample of 15 to 30 people will be needed for each of the 3 types of participants to achieve theoretical saturation, that is, the point at which data do not add any new properties to the theoretical concepts identified [55]. This saturation is fostered by comparing and contrasting emerging theoretical concepts with different empirical contexts [54,55].

##### Data Collection

Each participant will be met digitally on a secure platform (Teams) for a 60-90 minute semidirected, audio-recorded, and transcribed interview. This approach will help to recruit participants from across Québec, offer greater flexibility in the timing of interviews and ensure confidentiality. Based on the results of component 1 and the EVLN model, a semistructured interview guide will be developed for each type of participant, validated by 4 methodological experts (grounded theory) and content experts (health and management) and pretested with 3 graduate students in health and management. To promote theoretical saturation, the guides will be refined following the analysis of each interview. To characterize their profile, participants will also complete the sociodemographic section of the web-based questionnaire in component 1. Throughout data collection, memos (collection and analysis notes) will be used to (1) record preconceived notions (logbook), (2) clarify methodological choices (audit trail), and (3) outline emerging concepts and categories (data source). Memos will thus be used as a source and tool for analyzing the data and interpreting the results [51,54].

##### Tools

Semistructured interview guides will use open-ended questions to address change agent actions reported, factors involved in the emergence of fear of reprisal (clinical professionals and managers) or determination of wrongdoing (senior administrators and managers), and how that fear or determination emerged (process). For example, questions might be “What actions caused you to fear retaliation?” “Describe what led you, as a result of [this action], to determine that it was wrong”; “You told me that [this factor] led you to fear retaliation. What other [factors], if any, contributed to your fear? Explain”; “What actions did you determine to be objectionable? Explain.” These questions will elucidate the specific experience of participants while avoiding the use of preconceived categories [51]. Each guide will be developed according to the principles of Vermersch’s [56] explicitation technique. This interview technique aims to make participants’ experience of actions explicit, which reduces the risks of post hoc rationalization inherent in the retrospective nature of the method used in this study. Thus, respecting explicitation technique principles adds rigor to the chosen design and increases the credibility of the results.

##### Analyses

The analyses will be carried out independently for each type of participant. Sociodemographic data will be analyzed using descriptive statistics. Data from interviews will be transcribed using the Teams transcription function and transcripts will be verified against the audiotapes. All the data collected will be analyzed according to the constructivist perspective of grounded theory [51] and, to enrich the analysis [57], in relation to the EVLN model. This flexible and open-ended method makes it easier to understand complex processes [54] such as fear of reprisal and the determination of wrongdoing. The data will be analyzed using a constant comparison process [51]. The strategies used are (1) simultaneous data collection and analysis, (2) coding (open and then selective), (3) comparative analysis methods, (4) use of memos to facilitate analysis of the concepts under study, (5) theoretical sampling to refine theoretical constructs and saturate data, and (6) integration of theoretical structure (ie, updating conceptual links) to facilitate the emergence of a process model. Further, 3 models will be developed initially. The 2 models of the fear of reprisal process will then be compared to identify points of convergence and divergence, leading to an integrated model of fear of reprisal. The points of divergence and convergence between the integrated model of fear and the integrated model of determination of wrongdoing will also be documented to provide a comprehensive picture of these interrelated processes and form a final integrated process model.

#### Component 3: Legal and Ethical Analysis

To document the legal and ethical issues associated with the factors identified and the 2 processes described (objective 4), the situations described by the participants in the 3 groups will be analyzed from both a legal and an ethical viewpoint. Specifically, the legal analysis will include a collection of applicable legislation as well as jurisprudence from specialized labor and employment tribunals (notably arbitration awards) and disciplinary bodies (decisions of disciplinary committees and the Professions Tribunal) from the 2 leading legal databases (CanLII and Soquij). From an analysis of the circumstances in which courts and tribunals have concluded that the employer’s or the professional association’s sanctions were justified, it will be possible to identify situations in which fear of reprisal is legally founded. To understand the role played by the actors’ understanding or misunderstanding of the applicable law, these situations will then be compared to the factors involved in the emergence of fear of reprisal among clinical professionals and managers and the determination of wrongdoing among senior administrators and managers. On the ethical front, we will first analyze the same situations using Swisher and Royeen’s [58] model. This model aims to conceptually organize a situation’s ethical issues into 5 categories: dilemma, ambiguity, distress, temptation, or silence. The issues identified can then be deepened by mobilizing deductive ethical theories (deontology and consequentialism) and inductive ethical theories (ethics of care and axiology) using the Quadripartite Ethical Framework [59,60]. The results of this in-depth ethical analysis of situations mentioned by participants will provide clues to the ethical tools to be developed to improve how practicing and future professionals are trained.

### Ethical Considerations

The research protocol has been approved by the Research Ethics Board of the Université de Sherbrooke (Comité d’éthique de la recherche – Lettres et sciences humaines; #2022-3473/Carrier). All participants will be required to provide free and informed written consent [61]. Participants will be able to withdraw from this study at any time without any repercussions. They will also be informed of the confidential nature of the data collected and the procedures followed to ensure confidentiality and anonymity. The data collected, both electronically and on paper, will be secured (on a password-protected computer or in a locked fireproof filing cabinet at the Research Centre on Aging) and only the research assistant, research trainee, and principal investigator will have access to them. To ensure they are not lost, electronic data will also be stored on the Université de Sherbrooke’s secured institutional platform accessible only through institutional identification and password by the same team members.

## Results

This ongoing study began in June 2022 and will be conducted according to an optimal but realistic 5-year timeline that takes into account the uncertain context related to the COVID-19 pandemic (Table 1). Components 1 and 2 will be completed within a 4-year time frame (2022-2026) while component 3 will be completed in the final year. To ensure rapid knowledge mobilization, dissemination strategies will be employed by the entire team throughout this study.

**Table 1:** Tasks and timeline by component. This table describes the tasks and timeline for each component (C) of the 3-part sequential mixed-method design research over a 5-year period.

Task			C1^a^				C2^b^				C3^c^
Year			1		2		3		4	5	
**Ethics and C1 survey (quantitative)**											
	Ethical approval	✓		N/A^d^		N/A		N/A		N/A	
	Development of the questionnaire (collection from 3 sources; analysis, validation by Delphi, and pretest)	✓		N/A		N/A		N/A		N/A	
	Recruitment, data collection, and analysis	N/A		✓		N/A		N/A		N/A	
**C2 qualitative grounded theory**											
	Development of interview guides	N/A		N/A		✓		N/A		N/A	
	Recruitment, data collection, and analysis	N/A		N/A		✓		✓		N/A	
**C3 legal and ethical analysis**											
	Analysis of situations	N/A		N/A		N/A		N/A		✓	
	Dissemination and mobilization strategies	✓		✓		✓		✓		✓	

^a^C1: component 1.

^b^C2: component 2.

^c^C3: component 3.

^d^N/A: Not applicable.

## Discussion

### Strengths and Limitations

Building on established and new collaborations, the research team has all the interdisciplinary expertise required to conduct this study successfully and ensure effective and relevant dissemination and mobilization of the knowledge developed. In fact, a conscious effort has been made to secure complementary expertise and professional networks. Each profession concerned is represented, and each member has a high degree of credibility in his or her profession and strong ties to organizations related to that profession or to senior administrators and managers. This will undeniably facilitate the recruitment of participants, which is the main challenge of this study. The results should lead to a fleshed-out understanding of the fear of reprisal associated with change agent actions, a phenomenon that is apparently growing in HSS.

### Broad Implications

Using a sequential mixed-method design, this study will first establish the prevalence of fear of reprisal among clinical professionals and managers in the public HSS system who are particularly involved with the vulnerable population: nurses, occupational therapists, physical therapists, social workers, and speech therapists. Second, this research will identify the factors involved in the emergence of this fear among clinical professionals and managers and in the determination of wrongdoing by senior administrators and managers. Third, the emergence process of this fear among clinical professionals and managers and this determination by senior managers will be described. Finally, this study will document the legal and ethical issues associated with the factors identified and the processes described. This will provide a better understanding of the complex dynamic process leading to professionals’ and clinical managers’ fear of reprisal in their change agency actions and senior managers’ determination of wrongdoing.

The scientific knowledge developed in this study will help to prepare practicing and future professionals to work in real-world settings. In addition, this knowledge will be useful for university programs both in Québec and globally. These results will also be of interest to professional regulatory boards and associations, unions, and management associations, all of which are concerned with their members’ continuing education and quality of practice. Moreover, knowledge mobilization strategies will include the organization of forums (eg, training) to encourage the emergence of actions. Finally, the interest of researchers studying other public services potentially affected by fear of reprisal, such as education and the environment, will also be stimulated.

## Appendix

Multimedia Appendix 1 Peer review report by Social Sciences and Humanities Research Council of Canada (SSHRC). 435-23A (Multidisciplinary or interdisciplinary social sciences).

## Abbreviations

EVLN: Exit, Voice, Loyalty, Neglect
HSS: health and social service

## Data Availability

The research team abides by the Research Data Management Plan of the *Université de Sherbrooke*.
