# Supplementary material for: Fear of Reprisal and Change Agency in the Public Health and Social Service System: Protocol for a Sequential Mixed Methods Study
Source: JMIR Res Protoc. 2023 Sep 21;12:e48400. doi: 10.2196/48400 (PMC10556997; doi:10.2196/48400)
Supplement: Multimedia Appendix 1 [file resprot_v12i1e48400_app1.pdf]

www.sshrc-crsh.gc.ca

350 rue Albert, C.P. 1610
Ottawa ON K1P 6G4

350 Albert Street, P.O. Box 1610
Ottawa ON K1P 6G4

AVIS DE DÉCISION

Renseignements sur la demande

Candidate/Candidat : Annie Carrier

Établissement : Université de Sherbrooke

Occasion de financement : **subventions Savoir**

Numéro de la demande : 435-2022-1058

Volet choisi : VOLET B

Titre : La crainte de représailles : une étude mixte séquentielle pour optimiser la formation au rôle d'agent de changement dans le système public de services sociaux et de santé

Décision de financement : **Accordé**

Résultats de la demande

Vos notes au sein de votre comité:

Défi (40%):	4.25/ 6
Faisabilité (20%):	3.25/ 6
Capacité (40%):	4.88/ 6
Note finale (pondérée):	12.91/ 18

Une demande doit recevoir une note de 3.0 ou plus pour chacun des trois critères afin d'être recommandée pour un financement.

Rang: 12/31

Sextile dans le classement général du concours: Troisième

Résultats du comité

Nom du comité:
23A - Multidisciplinaire ou interdisciplinaire sciences sociales

Volet A

Demandes:	9
Subventions accordées:	6 (66.7%)
Subventions non accordées:	3 (33.3%)

Note de la dernière demande financée: 10.80

Volet B

Demandes:	31
Subventions accordées:	17 (54.8%)
Subventions non accordées:	14 (45.2%)

Note de la dernière demande financée: 12.28

Résultats du concours

Demandes :	1084
Subventions accordées :	560 (51.7%)
Subventions non accordées :	524 (48.3%)

Catégories sextiles :

Premier : 181 accordées / 0 non accordées

Deuxième : 181 accordées / 0 non accordées

Troisième : 181 accordées / 0 non accordées

Quatrième : 17 accordées / 164 non accordées

Cinquième et sixième : 0 accordées / 360 non accordées

Recommandation du comité

Le comité recommande que cette demande méritoire soit subventionnée avec une réduction budgétaire.
Réductions budgétaires recommandées dans le(s) domaine(s) de :
dépenses du personnel non-étudiant

Questions? Courriel: subventionssavoir@sshrc-crsh.gc.ca

SSHRC CRSH

**Comité:**	23A - Multidisciplinaire ou interdisciplinaire sciences sociales
**Candidat(e):**	Annie Carrier
**No de la demande:**	435-2022-1058
**No de l'évaluateur externe:**	1

Subventions Savoir

Formulaire d'évaluation externe

Évaluation

Instructions : Les évaluations externes ont pour objectif d'assister le comité dans ses délibérations. Le CRSH reconnaît votre expertise et vous sait gré du temps et des efforts consacrés à cette évaluation.

Étant donné la nature compétitive du processus de sélection, les candidats(es) bénéficieront de critiques constructives ou de suggestions sur la façon d'améliorer leur demande.

Le (la) candidat(e) recevra une copie intégrale de votre évaluation. Prière de n'y inscrire aucun renseignement personnel qui permettrait de vous identifier. Le cas échéant, le personnel du CRSH se réserve le droit de retirer ces renseignements.

Déclaration sur la confidentialité et sur les conflits d'intérêts

1) Les renseignements consignés dans les demandes sont protégés en vertu de la *Loi sur la protection des renseignements personnels* et ne sont accessibles aux appréciateurs externes que pour fins de l'examen des demandes. Je consens à considérer comme strictement confidentiels tous les documents provenant du dossier qui m'a été transmis par le Conseil. Après avoir répondu, positivement ou négativement, à la demande du Conseil, j'assurerai la destruction dudit matériel.

J'accepte	X

2) J'atteste que je ne suis pas en situation de conflit d'intérêts à l'égard du (des) candidat(s) dont j'évalue la demande.

J'accepte	X

En utilisant les lignes directrice dans le document ci-joint, veuillez évaluer chaque sous-critère ci-dessous et cochez la case appropriée. Expliquez brièvement votre réponse.

**Comité:**	23A - Multidisciplinaire ou interdisciplinaire sciences sociales
**Candidat(e):**	Annie Carrier
**No de la demande:**	435-2022-1058
**No de l'appréciateur:**	1

Partie 1 : Défi - Objectif et importance de la recherche

Sous-critères d'évaluation (Aucune pondération spécifique n'est attribuée aux sous-critères)	S/O	Insatisfaisant	Bon	Très Bon	Excellent
Originalité, importance et contribution prévue à l'avancement des connaissances				X	
Pertinence de la revue de littérature			X		
Pertinence du cadre ou de l'approche théorique				X	
Pertinence des méthodologies ou des approches				X	
Qualité de la formation et du mentorat offerts aux étudiants, aux chercheurs émergents et à d'autres personnes hautement qualifiées ainsi que des occasions qu'ils auront de contribuer à l'activité de recherche					X
Influence et impact probables des résultats du projet au sein et à l'extérieur de la communauté des chercheurs en sciences humaines					X

Veuillez expliquer brièvement votre réponse en terme de forces et faiblesses :

Forces :
Si le principe d'universalité de l'accès à des services de santé et des services sociaux de qualité à l'ensemble de la population s'applique encore aujourd'hui aux institutions du réseau public québécois, force est de constater que, dans les faits et pour diverses raisons, ce dernier n'est pas toujours respecté. Des situations d'inégalités sociales ou de santé, d'injustices ou d'exclusion de certaines catégories de personnes sont en effet encore trop régulièrement observées dans les établissements. Ces situations doivent alors être dénoncées et les droits des personnes qui les vivent défendus par les professionnels qui offrent les services. En ce sens, parce qu'il s'intéresse aux éléments pouvant nuire au rôle d'agent de changement des professionnels de la santé et des services sociaux, dont la crainte des représailles, le projet proposé s'insère dans les orientations et préoccupations actuelles du champ de la recherche scientifique et celles du milieu professionnel du secteur de la santé et des services sociaux. Il est possible en effet de constater qu'au cours des dernières années, en particulier cette dernière année et demi marquée par la pandémie mondiale, on a vu se multiplier la publication de travaux et d'études scientifiques sur les problèmes systémiques rencontrés dans de nombreuses institutions publiques, ainsi que de nombreuses sorties médiatiques de représentants syndicaux, de responsables d'ordres professionnels et d'intervenants professionnels dénonçant les conditions de travail causant de nombreux dilemmes éthiques chez le

personnel. L'ensemble des résultats découlant de cette recherche aura donc des retombées sur les plans scientifique, professionnel et académique.

L'originalité du projet repose en grande partie sur une approche multidisciplinaire où, au-delà des approches propres à chacun des champs disciplinaires représentés, le point commun aux divers professionnels, cadres et cadres supérieurs consiste en cette responsabilité organisationnelle partagée qui est d'agir pour le changement auprès des usagers. Rallier ainsi un grand nombre d'acteurs d'horizons professionnels différents autour de valeurs de justice sociale et d'égalité permet ainsi de transcender les divergences souvent inévitables au sein d'équipes multidisciplinaires.

Les choix méthodologiques apparaissent tout à fait en cohérence avec les objectifs visés. Le jumelage d'un devis qualitatif avec un devis quantitatif, déplié en trois volets distincts, permettra d'obtenir des données variées et pertinentes pour nourrir les réflexions sur le sujet et pour produire un corpus de connaissances scientifiques riches. Le volet 3 est particulièrement original, en ce qu'il permettra de documenter les enjeux de nature éthique et juridique à partir des situations vécues par les participants.

Les références utilisées sont pertinentes et concernent les différents volets de la recherche (problématique, cadre théorique, aspects méthodologiques), ce qui démontre que la chercheuse maîtrise bien le champ des connaissances relatives aux objets faisant partie de la demande. La bibliographie contient des travaux récents, issus du champ scientifique et quelques-uns sont de nature professionnelle, ainsi que des auteurs classiques dans le domaine (ex. Freire, Vermersch, etc.).

Il ne fait aucun doute que les étudiants qui se joindront à l'une ou l'autre des étapes de réalisation de la recherche sauront faire des apprentissages sur les divers aspects composant le processus de la recherche : conception d'instruments de collecte des données, réalisation d'entretiens de recherche, analyse de données qualitatives et quantitatives, participation à la rédaction d'articles scientifiques, communications dans des congrès, intégration à une équipe de chercheurs chevronnés. L'aspect multidisciplinaire est encore ici à souligner, car le fait d'impliquer des étudiants de divers horizons disciplinaires et professionnels ne pourra qu'enrichir les échanges et favoriser chez eux le développement d'habiletés à collaborer avec des acteurs provenant de champs différents, mais complémentaires.

Faiblesses :
La notion d'agent de changement mériterait d'être un peu plus explicitée, car, telle que présentée, elle ne semble pas faire de distinction entre les professionnels d'horizons divers. Selon les professions représentées, cette notion peut en effet se référer à de multiples significations. En quoi notamment tous les acteurs des champs disciplinaires visés se rejoignent-ils ou se distinguent-ils de cette définition? Comment s'opérationnalise ce rôle selon les professionnels? Il est mentionné que : (…) les AC utilisent une vaste gamme d'actions se déployant sur un continuum allant du clinique au sociétal (…) (p. 1) ». Quelles sont ces actions plus précisément? En quoi permettent-elles l'atteinte d'une plus grande justice sociale, considérant que ce ne sont pas toutes les professions qui incluent explicitement ce rôle à leur mandat?

Le lien entre la crainte de représailles chez les acteurs visés et les contextes ou facteurs organisationnels pouvant l'influencer n'est pas toujours évident à saisir. Une légère impression de décalage se remarque entre la problématique et les assises théoriques. Il semble que la problématique fait surtout état d'éléments ou de facteurs de nature individuelle, voire psychologique, alors que l'approche théorique préconisée semble davantage être de nature organisationnelle. La recension des écrits souligne que la crainte

**Comité:**	23A - Multidisciplinaire ou interdisciplinaire sciences sociales
**Candidat(e):**	Annie Carrier
**No de la demande:**	435-2022-1058
**No de l'appréciateur:**	1

de représailles peut découler d'éléments tels que l'interprétation du devoir de loyauté envers l'employeur, le mode de gestion « par la peur », la perception d'une action d'agent de changement répréhensible par les cadres supérieurs et hors-cadres. On indique plus loin qu'en formation, un accompagnement à la conscience critique permet une ouverture à la transformation personnelle. Or ces éléments témoignent surtout d'aspects relatifs à l'individu et moins à des éléments de contextes organisationnels. Les éléments ensuite abordés dans le cadre théorique prennent appui sur un modèle s'intéressant aux conditions de travail et à la satisfaction des professionnels au regard de ces conditions (modèle EVLN). Le fait que ces éléments ne sont pas identifiés avant accentue ce léger décalage entre la façon d'aborder la problématique et l'approche théorique préconisée pour l'analyser. Faire ressortir davantage, dans la recension des écrits, les liens avec les aspects organisationnels pouvant être liés, soutenir ou décourager la crainte de représailles chez les professionnels contribuerait certainement à réduire cet écart. Démontrer par exemple que, dans la littérature recensée, peu d'écrits ou de travaux ont porté sur les aspects organisationnels de la peur de représailles.

D'un point de vue méthodologique, bien que les choix s'avèrent pertinents au regard des objectifs visés, quelques incohérences relativement aux méthodes d'analyse qualitative sont perceptibles. C'est le cas notamment avec le choix de la méthode de théorisation enracinée (MTE) préconisée pour le deuxième volet. La distinction entre une MTE et une méthode d'analyse thématique n'est pas claire. En quoi s'agit-il davantage d'une MTE? Par ailleurs, quelle est la plus-value d'une méthode de théorisation enracinée dans ce cas-ci? Bref, le choix de cette méthode gagnerait à revu ou à être justifié davantage. Pour les « puristes » de cette méthode, il s'agit avant tout d'une approche générale inductive. Or, le principal outil de collecte des données inclura un guide d'entretien semi-structuré selon les principes de l'entretien d'explicitation de Vermersch, et les données qui seront récoltées seront ensuite mise en lien avec les éléments du modèle EVLN. Cette démarche laisse plutôt supposer une approche « semi-inductive » ou thématique. Ces outils sont en soi pertinents, mais comment seront-ils arrimés aux principes de la MTE?

Partie 2 : Faisabilité - Le plan visant l'atteinte de l'excellence

Sous-critères d'évaluation (Aucune pondération spécifique n'est attribuée aux sous-critères)	S/O	Insatisfaisant	Bon	Très Bon	Excellent
Pertinence du calendrier proposé et probabilité d'atteindre les objectifs de la recherche				X	
Expertise du candidat ou de l'équipe par rapport à la recherche proposée					X
Pertinence du budget demandé, justification des coûts proposés et, le cas échéant, obtention de contributions en espèces ou en nature provenant d'autres sources				X	

**Comité:**	23A - Multidisciplinaire ou interdisciplinaire sciences sociales
**Candidat(e):**	Annie Carrier
**No de la demande:**	435-2022-1058
**No de l'appréciateur:**	1

**Sous-critères d'évaluation (Aucune pondération spécifique n'est attribuée aux sous-critères)**	**S/O**	**Insatisfaisant**	**Bon**	**Très Bon**	**Excellent**
Qualité et pertinence des plans de mobilisation des connaissances, y compris la diffusion des connaissances, les échanges et la collaboration efficaces avec les intervenants au sein et à l'extérieur de la communauté des chercheurs s'il y a lieu				X	

Veuillez expliquer brièvement votre réponse en terme de forces et faiblesses :

Forces :

Les contributions de la candidate sont relatives à la conceptualisation du rôle d'agent de changement des professionnels du secteur de la santé et des services sociaux. Elle a développé un modèle permettant de planifier les actions des agents de changement, lequel est à la fois pertinent au champ professionnel et à celui de la formation initiale des futurs professionnels sociosanitaires. En cohérence avec ses propres objets de recherche, le présent projet s'insère directement dans la continuité de ses travaux, ce qui lui permettra de développer de nouvelles connaissances et de les ajouter à celles composant son corpus déjà constitué sur le sujet. Professeure universitaire depuis 2017 à la Faculté de médecine et des sciences de la santé de l'Université de Sherbrooke, elle a depuis obtenu des subventions d'organismes subventionnaires reconnus (FRQS, CRSH) à titre de chercheuse principale et de cochercheuse. Les projets de recherche qu'elle conduit ou auxquels elle est associée portent sur divers aspects des pratiques professionnelles d'agents œuvrant dans le secteur de la santé et des services sociaux, se situant ainsi dans la prolongation de ses préoccupations de recherche.

Les activités de recherche de la candidate ne se centrent pas uniquement sur le développement des connaissances par la réalisation de recherche sur ses objets d'intérêts, mais se manifestent également par des activités de diffusion des connaissances. Celles-ci prennent surtout la forme d'articles scientifiques rédigés à titre de première auteure et évalués par les pairs et donc destinés à la communauté scientifique.

La candidate, ainsi que les chercheuses et cochercheurs associés à ce projet, ont une expérience non négligeable d'encadrement d'étudiants de cycles supérieurs, de stagiaires ou encore d'étudiants en initiation à la recherche. Ceci constitue un appui indéniable pour le soutien et la formation nécessaires aux étudiants qui prendront part à ce projet.

Faiblesses :
Le volet mobilisation des connaissances semble surtout se focaliser sur les connaissances scientifiques. Il serait intéressant de faire ressortir davantage les activités de diffusion destinées à un public élargi, hors de la communauté scientifique, par exemple les professionnels du secteur de la santé et des services sociaux, les ordres ou associations professionnels, etc.

Partie 3 : Capacité - Potentiel de réussite

**Comité:**	23A - Multidisciplinaire ou interdisciplinaire sciences sociales
**Candidat(e):**	Annie Carrier
**No de la demande:**	435-2022-1058
**No de l'appréciateur:**	1

Dans le cas d'une équipe de recherche, évaluez les résultats de recherche de chaque membre de l'équipe (n'incluez pas les collaborateurs).

Dans votre évaluation, répondez aux critères suivants en considérant l'étape de carrière du candidat et les membres de l'équipe, le cas échéant:

Sous-critères d'évaluation (Aucune pondération spécifique n'est attribuée aux sous-critères)	S/O	Insatisfaisant	Bon	Très Bon	Excellent
Qualité, quantité et importance de l'expérience antérieure, des réalisations artistiques ou des publications du candidat et des cocandidats, s'il y a lieu, en fonction du rôle joué au sein du projet et du stade d'évolution de sa carrière				X	
Démonstration d'activités de mobilisation des connaissances (p.ex. films, prestations, rapports commandés, synthèses des connaissances, expérience de collaboration ou d'interaction avec des intervenants, contributions aux débats publics et médiatiques) et de l'impact sur les pratiques professionnelles, les politiques et services sociaux, etc.				X	
Qualité et quantité des contributions antérieures apportées à la formation et au mentorat d'étudiants, de chercheurs postdoctoraux et d'autres personnes hautement qualifiées				X	

Veuillez expliquer brièvement votre réponse en terme de forces et faiblesses :

Forces :
Les contributions de la candidate sont relatives à la conceptualisation du rôle d'agent de changement des professionnels du secteur de la santé et des services sociaux. Elle a développé un modèle permettant de planifier les actions des agents de changement, lequel est à la fois pertinent au champ professionnel et à celui de la formation initiale des futurs professionnels sociosanitaires. En cohérence avec ses propres objets de recherche, le présent projet s'insère directement dans la continuité de ses travaux, ce qui lui permettra de développer de nouvelles connaissances et de les ajouter à celles composant son corpus déjà constitué sur le sujet. Professeure universitaire depuis 2017 à la Faculté de médecine et des sciences de la santé de l'Université de Sherbrooke, elle a depuis obtenu des subventions d'organismes subventionnaires reconnus (FRQS, CRSH) à titre de chercheuse principale et de cochercheuse. Les projets de recherche qu'elle conduit ou auxquels elle est associée portent sur divers aspects des pratiques professionnelles d'agents œuvrant

dans le secteur de la santé et des services sociaux, se situant ainsi dans la prolongation de ses préoccupations de recherche.

Les activités de recherche de la candidate ne se centrent pas uniquement sur le développement des connaissances par la réalisation de recherches sur ses objets d'intérêts, mais se manifestent également par des activités de diffusion des connaissances. Celles-ci prennent surtout la forme d'articles scientifiques rédigés à titre de première auteure et évalués par les pairs et donc destinés à la communauté scientifique.

La candidate, ainsi que les cochercheuses et cochercheurs associés à ce projet, ont une expérience non négligeable d'encadrement d'étudiants de cycles supérieurs, de stagiaires ou encore d'étudiants en initiation à la recherche. Ceci constitue un appui indéniable pour le soutien et la formation nécessaires aux étudiants qui prendront part à ce projet.

Faiblesses :
Le volet mobilisation des connaissances semble surtout se focaliser sur les connaissances scientifiques. Il serait intéressant de faire ressortir davantage les activités de diffusion destinées à un public élargi, hors de la communauté scientifique, par exemple les professionnels du secteur de la santé et des services sociaux, les ordres ou associations professionnels, etc.

Partie 4 : Commentaire additionnel

Si vous avez des commentaires concernant le budget ou autres aspects de la demande, veuillez les indiquer ici.

PROTECTED B WHEN COMPLETED / PROTÉGÉ B UNE FOIS REMPLI

SSHRC CRSH

**Comité:**	23A - Multidisciplinaire ou interdisciplinaire sciences sociales
**Candidat(e):**	Annie Carrier
**No de la demande:**	435-2022-1058
**No de l'évaluateur externe:**	3

Subventions Savoir

Formulaire d'évaluation externe

Évaluation

Instructions : Les évaluations externes ont pour objectif d'assister le comité dans ses délibérations. Le CRSH reconnaît votre expertise et vous sait gré du temps et des efforts consacrés à cette évaluation.

Étant donné la nature compétitive du processus de sélection, les candidats(es) bénéficieront de critiques constructives ou de suggestions sur la façon d'améliorer leur demande.

Le (la) candidat(e) recevra une copie intégrale de votre évaluation. Prière de n'y inscrire aucun renseignement personnel qui permettrait de vous identifier. Le cas échéant, le personnel du CRSH se réserve le droit de retirer ces renseignements.

Déclaration sur la confidentialité et sur les conflits d'intérêts

1) Les renseignements consignés dans les demandes sont protégés en vertu de la *Loi sur la protection des renseignements personnels* et ne sont accessibles aux appréciateurs externes que pour fins de l'examen des demandes. Je consens à considérer comme strictement confidentiels tous les documents provenant du dossier qui m'a été transmis par le Conseil. Après avoir répondu, positivement ou négativement, à la demande du Conseil, j'assurerai la destruction dudit matériel.

J'accepte	X

2) J'atteste que je ne suis pas en situation de conflit d'intérêts à l'égard du (des) candidat(s) dont j'évalue la demande.

J'accepte	X

En utilisant les lignes directrice dans le document ci-joint, veuillez évaluer chaque sous-critère ci-dessous et cochez la case appropriée. Expliquez brièvement votre réponse.

**Comité:**	23A - Multidisciplinaire ou interdisciplinaire sciences sociales
**Candidat(e):**	Annie Carrier
**No de la demande:**	435-2022-1058
**No de l'appréciateur:**	3

Partie 1 : Défi - Objectif et importance de la recherche

Sous-critères d'évaluation (Aucune pondération spécifique n'est attribuée aux sous-critères)	S/O	Insatisfaisant	Bon	Très Bon	Excellent
Originalité, importance et contribution prévue à l'avancement des connaissances				X	
Pertinence de la revue de littérature				X	
Pertinence du cadre ou de l'approche théorique				X	
Pertinence des méthodologies ou des approches					X
Qualité de la formation et du mentorat offerts aux étudiants, aux chercheurs émergents et à d'autres personnes hautement qualifiées ainsi que des occasions qu'ils auront de contribuer à l'activité de recherche				X	
Influence et impact probables des résultats du projet au sein et à l'extérieur de la communauté des chercheurs en sciences humaines					X

Veuillez expliquer brièvement votre réponse en terme de forces et faiblesses :

La problématique de recherche, bien articulée, est pertinente et d'actualité. Les objectifs sont cohérents et bien justifiés par la recension des écrits. La méthodologie (devis mixte séquentiel) appuie chacun des objectifs de l'étude. L'étude s'appuie sur une perspective constructiviste et est guidée par le modèle Exit, Voice, Loyalty, Neglect proposé par Rusbult et collègues. Ce modèle est mobilisé dans les phases préparatoires et empiriques/analytiques de l'étude. Il est surprenant que des concepts-clés connexes au phénomène de représailles ou de craintes de représailles ne soient pas théorisés, ou même abordés dans la conceptualisation du phénomène-problème. Des représailles incluent régulièrement des pratiques de réprimandes (parfois devant les pairs), du harcèlement, de l'intimidation ou des sanctions arbitraires (sans 'enquête') au centre desquelles des abus de pouvoir et d'autorité sont probants. Sachant cela, une prise en compte et une théorisation des dynamiques de pouvoir ou de pratiques d'injustice en jeu (souvent cristalisées dans les structures et cultures organisationnelles) deviennent toute indiquées. Je m'étonne d'autant plus de cette omission considérant que les chercheurs abordent ce projet dans une optique de justice sociale - un champ dans lequel les concepts de justice et de pouvoir sont pourtant centraux.
Compte tenu que les professions ciblées pour ce projet sont très majoritairement féminines, une considération spéciale doit impérativement être accordée aux dynamiques de genre et, surtout, de socialisations professionnelles fondées sur le genre, qui ont été largement

documentées dans les écrits théoriques et empiriques. L'équipe bénéficierait d'une chercheuse ou d'un chercheur pouvant contribuer une perspective (ex: féministe, intersectionnelle...) à ce projet.
Ce projet prend au sérieux le rôle et la responsabilité des chercheurs en matière de formation des étudiants et donne à ces derniers un rôle central. L'équipe inclut également un bon mélange de chercheurs chevronnés et plus juniors, issus de diverses disciplines, et dans une proportion appropriée hommes-femmes.
Les résultats escomptés feront une contribution intéressante et utile aux connaissances en sciences humaines, sociales et de la santé.

Partie 2 : Faisabilité - Le plan visant l'atteinte de l'excellence

Sous-critères d'évaluation (Aucune pondération spécifique n'est attribuée aux sous-critères)	S/O	Insatisfaisant	Bon	Très Bon	Excellent
Pertinence du calendrier proposé et probabilité d'atteindre les objectifs de la recherche					X
Expertise du candidat ou de l'équipe par rapport à la recherche proposée					X
Pertinence du budget demandé, justification des coûts proposés et, le cas échéant, obtention de contributions en espèces ou en nature provenant d'autres sources			X		
Qualité et pertinence des plans de mobilisation des connaissances, y compris la diffusion des connaissances, les échanges et la collaboration efficaces avec les intervenants au sein et à l'extérieur de la communauté des chercheurs s'il y a lieu				X	

Veuillez expliquer brièvement votre réponse en terme de forces et faiblesses :

Le calendrier proposé est cohérent compte tenu du contexte actuel. Le plan de mobilisation des connaissances est solide. L'un de ses éléments (projet de transfert des connaissances requis dans certains cours à l'U de Sherbrooke) manque de précision et de clarté, et quelques autres éléments semblent flous (ex: transfert des connaissances lié au Volet 1) mais cela n'affecte pas le plan d'ensemble outre mesure.
Le projet s'inscrit en continuité avec les travaux récents de la plupart des membres et tout particulièrement avec ceux de la chercheuse principale. Cette dernière présente un profil adapté à la nature, à l'étendue et à la complexité du projet.
Les chercheurs rassemblés pour ce projet présentent un bon mélange interdisciplinaire d'expertises complémentaires. Tel que mentionné précédemment, l'équipe devrait impérieusement se doter d'une chercheuse ou d'un chercheur pouvant contribuer - de manière substantielle - une perspective genrée et/ou intersectionnelle qui est indispensable à ce

projet.
Le budget est élevé tout en s'échelonnant sur 5 ans. La grosse majorité des fonds est dédiée à des étudiants et stagiaires. Ceci est appréciable. Je souligne toutefois ma préoccupation de laisser certains éléments-clés de l'étude entre les mains d'étudiants gradués qui, selon moi, ne s'y prêtent pas entièrement. Le projet prévoit notamment la conduite des entrevues du volet qualitatif par des étudiants gradués. Considérant que ce sujet est sensible et qu'un tel entretien pourrait susciter des réactions émotionnelles fortes de la part des participants, ce type d'entretien doit être mené par un chercheur qui a de l'expérience avec ce type de situations et qui sait comment les aborder et les gérer. Les chercheurs devraient considérer mener ces entretiens eux-mêmes ou du moins en tandem avec des étudiants avant de leur en remettre l'entière responsabilité.

Partie 3 : Capacité - Potentiel de réussite

Dans le cas d'une équipe de recherche, évaluez les résultats de recherche de chaque membre de l'équipe (n'incluez pas les collaborateurs).

Dans votre évaluation, répondez aux critères suivants en considérant l'étape de carrière du candidat et les membres de l'équipe, le cas échéant:

Sous-critères d'évaluation (Aucune pondération spécifique n'est attribuée aux sous-critères)	S/O	Insatisfaisant	Bon	Très Bon	Excellent
Qualité, quantité et importance de l'expérience antérieure, des réalisations artistiques ou des publications du candidat et des cocandidats, s'il y a lieu, en fonction du rôle joué au sein du projet et du stade d'évolution de sa carrière				X	
Démonstration d'activités de mobilisation des connaissances (p.ex. films, prestations, rapports commandés, synthèses des connaissances, expérience de collaboration ou d'interaction avec des intervenants, contributions aux débats publics et médiatiques) et de l'impact sur les pratiques professionnelles, les politiques et services sociaux, etc.				X	
Qualité et quantité des contributions antérieures apportées à la formation et au mentorat d'étudiants, de chercheurs postdoctoraux et d'autres personnes hautement qualifiées					X

Comité: 23A - Multidisciplinaire ou interdisciplinaire sciences sociales
Candidat(e): Annie Carrier
No de la demande: 435-2022-1058
No de l'appréciateur: 3

Veuillez expliquer brièvement votre réponse en terme de forces et faiblesses :

J'estime que l'équipe additionnée d'une expertise féministe/intersectionnelle, tel que mentionné précédemment, a le potentiel de mener à bien ce projet et de produire des analyses de qualité.

Partie 4 : Commentaire additionnel

Si vous avez des commentaires concernant le budget ou autres aspects de la demande, veuillez les indiquer ici.

SSHRC ≡ CRSH

**Comité:**	23A - Multidisciplinaire ou interdisciplinaire sciences sociales
**Candidat(e):**	Annie Carrier
**No de la demande:**	435-2022-1058
**No de l'évaluateur externe:**	4

Subventions Savoir

Formulaire d'évaluation externe

Évaluation

Instructions : Les évaluations externes ont pour objectif d'assister le comité dans ses délibérations. Le CRSH reconnaît votre expertise et vous sait gré du temps et des efforts consacrés à cette évaluation.

Étant donné la nature compétitive du processus de sélection, les candidats(es) bénéficieront de critiques constructives ou de suggestions sur la façon d'améliorer leur demande.

Le (la) candidat(e) recevra une copie intégrale de votre évaluation. Prière de n'y inscrire aucun renseignement personnel qui permettrait de vous identifier. Le cas échéant, le personnel du CRSH se réserve le droit de retirer ces renseignements.

Déclaration sur la confidentialité et sur les conflits d'intérêts

1) Les renseignements consignés dans les demandes sont protégés en vertu de la *Loi sur la protection des renseignements personnels* et ne sont accessibles aux appréciateurs externes que pour fins de l'examen des demandes. Je consens à considérer comme strictement confidentiels tous les documents provenant du dossier qui m'a été transmis par le Conseil. Après avoir répondu, positivement ou négativement, à la demande du Conseil, j'assurerai la destruction dudit matériel.

J'accepte	X

2) J'atteste que je ne suis pas en situation de conflit d'intérêts à l'égard du (des) candidat(s) dont j'évalue la demande.

J'accepte	X

En utilisant les lignes directrice dans le document ci-joint, veuillez évaluer chaque sous-critère ci-dessous et cochez la case appropriée. Expliquez brièvement votre réponse.

**Comité:**	23A - Multidisciplinaire ou interdisciplinaire sciences sociales
**Candidat(e):**	Annie Carrier
**No de la demande:**	435-2022-1058
**No de l'appréciateur:**	4

Partie 1 : Défi - Objectif et importance de la recherche

Sous-critères d'évaluation (Aucune pondération spécifique n'est attribuée aux sous-critères)	S/O	Insatisfaisant	Bon	Très Bon	Excellent
Originalité, importance et contribution prévue à l'avancement des connaissances			X		
Pertinence de la revue de littérature			X		
Pertinence du cadre ou de l'approche théorique				X	
Pertinence des méthodologies ou des approches				X	
Qualité de la formation et du mentorat offerts aux étudiants, aux chercheurs émergents et à d'autres personnes hautement qualifiées ainsi que des occasions qu'ils auront de contribuer à l'activité de recherche					X
Influence et impact probables des résultats du projet au sein et à l'extérieur de la communauté des chercheurs en sciences humaines				X	

Veuillez expliquer brièvement votre réponse en terme de forces et faiblesses :

L'intérêt pour le rôle d'agents de changement (AC) des professionnels des services sociaux et de santé (SSS) est intéressant et apparaît pertinent, tant d'un point de vue clinique que scientifique. La problématique et la recension des écrits offrent un panorama des enjeux. Par contre, certaines orientations théoriques nous semblent mal justifiés, notamment, le choix de se centrer sur l'hypothèse principale de la peur de représailles. Pourquoi ce centrer spécifiquement sur cette hypothèse? Quelles étaient les autres hypothèses soulevées par la recherche citée en référence et pourquoi privilégier celle-ci? Quelles sont les connaissances, à l'échelle internationale, de la notion de la peur de représailles dans les SSS? Cette démonstration n'étant pas faite; on doit embarquer d'emblée dans la proposition qui opérationnalise la peur des représaille comme étant l'enjeu central des réticences à jouer le rôle d'AC.
Le cadre théorique et méthodologique nous apparaissent très originaux et pertinents pour la démarche et consituent une force de la proposition.

Partie 2 : Faisabilité - Le plan visant l'atteinte de l'excellence

Comité: 23A - Multidisciplinaire ou interdisciplinaire sciences sociales
Candidat(e): Annie Carrier
No de la demande: 435-2022-1058
No de l'appréciateur: 4

Sous-critères d'évaluation (Aucune pondération spécifique n'est attribuée aux sous-critères)	S/O	Insatisfaisant	Bon	Très Bon	Excellent
Pertinence du calendrier proposé et probabilité d'atteindre les objectifs de la recherche				X	
Expertise du candidat ou de l'équipe par rapport à la recherche proposée					X
Pertinence du budget demandé, justification des coûts proposés et, le cas échéant, obtention de contributions en espèces ou en nature provenant d'autres sources				X	
Qualité et pertinence des plans de mobilisation des connaissances, y compris la diffusion des connaissances, les échanges et la collaboration efficaces avec les intervenants au sein et à l'extérieur de la communauté des chercheurs s'il y a lieu			X		

Veuillez expliquer brièvement votre réponse en terme de forces et faiblesses :

Les forces: La proposition est très explicite par rapport au calendrier et aux étapes de réalisation de la recherche. À cet égard, l'évaluateur peut suivre avec précision l'ensemble des étapes et évaluer leur pertinence. L'expertise et l'engagement des cochercheurs sont bien mis en évidence et apparaîssent fort utiles pour l'atteinte des objectifs du projet. Le budget consacre la très grande majorité des fonds à la formation d'étudiant.es et à cet égard, remplit la mission de formation.

Les limites: Le calendrier séquencé laisse entrevoir certaines difficultés, notamment, le fait que le volet qualitatif, qui comprend jusqu'à 90 entretiens semi-dirigés de 60-90 minutes, sera concentré sur 2 ans. Cela nous apparaît assez serré.
Le plan de mobilisation et transfert des connaissances est intéressant, mais nous aurions aimé avoir plus de précision sur les stratégies dites intégrées. Par exemple, dans le volet 1, on parle de "séances" avec une pluralité d'acteurs (ordres et associations professionnelles, syndicats, asso. de gestionnaires, cinq établissements SSS): on n'a pas d'information sur ce qu'est une "séance": but et objectifs, personnes visées, durée, etc.

Partie 3 : Capacité - Potentiel de réussite

Dans le cas d'une équipe de recherche, évaluez les résultats de recherche de chaque membre de l'équipe (n'incluez pas les collaborateurs).

Dans votre évaluation, répondez aux critères suivants en considérant l'étape de carrière du candidat et les membres de l'équipe, le cas échéant:

**Comité:**	23A - Multidisciplinaire ou interdisciplinaire sciences sociales
**Candidat(e):**	Annie Carrier
**No de la demande:**	435-2022-1058
**No de l'appréciateur:**	4

Sous-critères d'évaluation (Aucune pondération spécifique n'est attribuée aux sous-critères)	S/O	Insatisfaisant	Bon	Très Bon	Excellent
Qualité, quantité et importance de l'expérience antérieure, des réalisations artistiques ou des publications du candidat et des cocandidats, s'il y a lieu, en fonction du rôle joué au sein du projet et du stade d'évolution de sa carrière					X
Démonstration d'activités de mobilisation des connaissances (p.ex. films, prestations, rapports commandés, synthèses des connaissances, expérience de collaboration ou d'interaction avec des intervenants, contributions aux débats publics et médiatiques) et de l'impact sur les pratiques professionnelles, les politiques et services sociaux, etc.				X	
Qualité et quantité des contributions antérieures apportées à la formation et au mentorat d'étudiants, de chercheurs postdoctoraux et d'autres personnes hautement qualifiées					X

Veuillez expliquer brièvement votre réponse en terme de forces et faiblesses :

L'équipe de recherche offre une riche expérience et des expertises complémentaires qui laissent entrevoir un fort potentiel à réaliser les objectifs de l'étude.

Partie 4 : Commentaire additionnel

Si vous avez des commentaires concernant le budget ou autres aspects de la demande, veuillez les indiquer ici.

Le budget prévoit 194 200$ pour l'embauche d'un agent de recherche. Aucune précision par rapport aux rôles et responsabilités de cette n'est fournie. Étant donné le nombre important de personnel de recherche qui sera impliqué à travers le projet, le rôle de cette personne devrait être précisé.

Conseil de recherches en sciences humaines du Canada
Évaluation du comité
Concours d’octobre 2021 des subventions Savoir

**Comité :**	435-23A (Multidisciplinaire ou interdisciplinaire sciences sociales)
**Dossier :**	435-2022-1058
**Nom du candidat :**	Annie Carrier
**Titre de la demande :**	La crainte de représailles : une étude mixte séquentielle pour optimiser la formation au rôle d'agent de changement dans le système public de services sociaux et de santé

Défi - Objectif et importance de l’activité de recherche (40 p. 100)

Sous-critères d’évaluation (Aucune pondération spécifique n’est attribuée aux sous-critères)	S/O	Insatisfaisant	Satisfaisant à bon	Bon à très bon	Très bon à excellent
		< 3.0	3.0-3.9	4.0-4.9	5.0-6.0
originalité, importance et contribution prévue à l’avancement des connaissances				X	
pertinence de la revue de la littérature			X		
pertinence du cadre ou de l’approche théorique			X		
pertinence des méthodologies ou des approches				X	
qualité de la formation et du mentorat offerts aux étudiants, aux chercheurs émergents et à d'autres personnes hautement qualifiées et possibilités qu’ils auront de contribuer à l’activité de recherche				X	
influence et impact probables des résultats du projet au sein et à l’extérieur de la communauté de recherche en sciences humaines				X	

Faisabilité - Plan visant l’atteinte de l’excellence (20 p. 100)

Sous-critères d’évaluation (Aucune pondération spécifique n’est attribuée aux sous-critères)	S/O	Insatisfaisant	Satisfaisant à bon	Bon à très bon	Très bon à excellent
		< 3.0	3.0-3.9	4.0-4.9	5.0-6.0
pertinence du calendrier proposé et probabilité d'atteindre les objectifs de la recherche			X		
Expertise du candidat ou de l’équipe par rapport à la recherche proposée				X	
pertinence du budget demandé, justification des coûts proposés et, le cas échéant, obtention de contributions en espèces ou en nature provenant d’autres sources		X			

Conseil de recherches en sciences humaines du Canada
Évaluation du comité
Concours d’octobre 2021 des subventions Savoir

Sous-critères d’évaluation (Aucune pondération spécifique n’est attribuée aux sous-critères)	S/O	Insatisfaisant	Satisfaisant à bon	Bon à très bon	Très bon à excellent
		< 3.0	3.0-3.9	4.0-4.9	5.0-6.0
qualité et pertinence des plans de mobilisation des connaissances, y compris la diffusion des connaissances, les échanges et la collaboration efficaces avec les parties prenantes au sein et à l’extérieur du milieu de la recherche s’il y a lieu				X	

Capacité - Potentiel de réussite (40 p. 100)

Sous-critères d’évaluation (Aucune pondération spécifique n’est attribuée aux sous-critères)	S/O	Insatisfaisant	Satisfaisant à bon	Bon à très bon	Très bon à excellent
		< 3.0	3.0-3.9	4.0-4.9	5.0-6.0
qualité, quantité et importance de l’expérience antérieure, des réalisations artistiques et (ou) des publications du candidat et des cocandidats, s’il y a lieu, en fonction du rôle joué au sein du projet et du stade d’évolution de leur carrière					X
démonstration d’activités de mobilisation des connaissances antérieures (p.ex. films, prestations, rapports commandés, synthèses des connaissances, expérience de collaboration ou d’interaction avec des parties prenantes, contributions aux débats publics et médiatiques) et de l’impact sur les pratiques professionnelles, les politiques sociales, les services sociaux, etc.				X	
Qualité et quantité des contributions antérieures à la formation et au mentorat d’étudiants, de chercheurs de niveau postdoctoral et d’autres personnes hautement qualifiées					X

Commentaires :

Le comité estime que la revue de littérature pourrait être mieux développée, notamment en ce qui concerne les agents de changement, les représailles et les relations de pouvoir.

Budget :

Le comité considère que le montant demandé pour l'agent de recherche aurait pu être mieux justifié.
